# Mantra meditation in sport: a scoping review of its use as a mental training and coping strategy for psychological well-being

**DOI:** 10.1080/00049530.2026.2700236

**Published:** 2026-07-13

**Authors:** Siqi Liu, Young-Eun Noh, Jeonghyo Kim

**Affiliations:** a School of Physical Education, Neijiang Normal University, Neijiang City, Sichuan Province, China; b Faculty of Sports and Exercise Science, University of Malaya, Kuala Lumpur, Malaysia; c Department of Physical Education, Seoul National University, Seoul, South Korea

**Keywords:** Mental health, sport injury, anxiety, mental toughness, spirituality

## Abstract

**Objective:**

The purpose of this study was to conduct a scoping review to map the existing literature on the use of mantra meditation in sport contexts, clarify its conceptual distinctions from related constructs such as self-talk, and identify directions for future research.

**Method:**

This study employed a comprehensive literature search, combining electronic database searches (including Web of Science, Psychology & Behavioural Sciences Collection, PubMed, SPORTDiscus, and Scopus) with manual searches, following the guidelines of the Preferred Reporting Items for Systematic Reviews and Meta-Analyses extension for scoping reviews.

**Results:**

The search initially identified 17,346 articles electronically and 324 articles manually. After a four-step screening process – duplicate removal, primary screening, secondary screening, and expert consensus – a total of four articles were initially included, with two additional articles added through a manual search by the first author, resulting in six articles for review. These studies were categorised into two main domains: mantra meditation as a mental training strategy and mantra meditation as a coping strategy.

**Conclusions:**

The findings provide preliminary insights into how athletes describe using mantra meditation in sport contexts. Across the included studies, athletes reported using mantras to regulate attention, maintain pace or rhythm, cope with fatigue and psychological stress, and support their sense of spiritual meaning and mental well-being. However, the limited number of available studies highlights the early stage of research in this area. Further high-quality empirical and intervention-based research is needed to clarify the mechanisms and potential effectiveness of mantra meditation when applied as a mental training strategy and as a coping strategy in sport contexts.

## KEY POINTS

What is already known about this topic:

(1) Psychological strategies are widely used in sport to enhance performance, support mental well-being, and facilitate athletes’ adjustment following injury.

(2) Mindfulness-based interventions have shown beneficial effects for athletes, including injured athletes, particularly in relation to stress regulation and psychological adaptation.

(3) Sleep disturbance, attentional deficits, and emotional dysregulation are common psychological challenges associated with sports injury and rehabilitation.

What this study adds:

(1) This study provides the first scoping review of the empirical literature on mantra meditation in sport, mapping how it has been conceptualised both as a mental training strategy and as a coping strategy.

(2) Findings suggest that athletes reported using mantras to regulate attention, maintain pace or rhythm, cope with fatigue and psychological stress, and support psychological well-being during sport participation.

(3) Existing studies indicate that mantra practices have been used by athletes across different sport contexts, including training and competition.

A mantra is a phrase or sound that has been repeated and sanctified over time within a spiritual tradition (Oman, 2025), sometimes referred to as mantra repetition or mantra chanting (Bormann et al., 2020). The applicability of the mantra is widespread. For instance, Raghuwanshi et al. (2022) found that Vedic chanting, a mantra originating in India, can improve sleep quality in drivers and positively affect their reaction time and fatigue levels. In addition, Corney et al. (2024) found that mantra repetition could reduce anxiety, stress, and depression levels in adults with autism.

Given the broad and positive role of mantra-based techniques, the United States Department of Veterans Affairs developed a mantra repetition programme. This programme provides a standardised mantra repetition guideline, guiding the individual on aspects such as duration, method, and frequency of practice, ensuring consistency and effectiveness in its application (Oman et al., 2022). Based on this standardised guideline, a substantial body of empirical research has implemented this programme with veterans and demonstrated that mantra meditation appears to have a beneficial impact on clinical outcomes for those with Post-Traumatic Stress Disorder (PTSD, Hassan et al., 2024). Beyond this specific population and condition, recent studies have also applied the mantra repetition programme to cancer patients, individuals with impaired fasting glucose, and those with substance use disorder, showing effectiveness in reducing perceived stress and alleviating substance use-related issues (Hassan et al., 2024; Hulett et al., 2024).

Mantra can also serve as a core technique within meditation, commonly referred to as mantra meditation (Ospina et al., 2007). Existing research outside sport has associated mantra meditation with psychological, cognitive, physiological, and spiritual outcomes, including well-being, quality of life, concentration, perceived stress, fatigue, sleep quality, and spiritual well-being (Henneghan et al., 2021; Hulett et al., 2024; Kelsven et al., 2024; Kostovich et al., 2021; Raghuwanshi et al., 2022; Singh et al., 2025). For example, Ventura et al. (2024) suggested that mantra meditation may involve shorter temporal windows of brain activity than some other forms of meditation, which may be relevant to attentional processes. However, despite this broader literature, the application of mantra meditation in sport remains limited and requires clearer conceptual and empirical mapping.

In competitive sports, the application of psychological strategies is crucial, whether to support athletes returning from injury (Annear et al., 2019) or to enhance performance in high-level competitions (Einarsson et al., 2020) and mental well-being (Zilli et al., 2023). Among various psychological strategies, mantra meditation has shown potential benefits in non-athletic populations.

## Conceptualization of mantra meditation

Mantra meditation refers to a practice in which individuals repeatedly vocalise or silently repeat a word or phrase (i.e., a mantra) in order to sustain attention or enter a meditative state. The repeated phrase may not carry a clear semantic or task-related meaning; rather, its use typically serves to establish a rhythmic focus of attention or to fulfil spiritual or existential needs, rather than to convey motivational or instructional information (Oman, 2025).

This absence of explicit semantic or task-related meaning highlights a key distinction from self-talk. Self-talk refers to an internally or externally articulated dialogue in which the sender and receiver of the message are the same person. Such statements are linguistically identifiable and typically contain explicit semantic content that is used to regulate cognition, emotion, or behaviour (e.g., motivational, instructional, or evaluative statements) (Van Raalte et al., 2015).

Furthermore, the universal applicability and low theoretical demand of mantra meditation also distinguish it from repetitive prayer practices in sport contexts (Bormann et al., 2020). Repetitive prayer practices constitute a form of religious or spiritual activity in which individuals repeatedly recite or silently repeat fixed prayers, sacred phrases, or scriptural passages (Noh & Liu, 2026). These practices typically follow established traditions of religious worship and are intended to express faith, cultivate spiritual focus, or facilitate a perceived connection with a sacred presence (Noh & Shahdan, 2022).

Mantra meditation also differs from traditional psychological coping strategies, such as imagery. Imagery is defined as an experience-construction process generated from memory information, characterised by quasi-sensorial, quasi-perceptual, and quasi-affective features (White & Hardy, 1998). This process is voluntarily controlled by the individual and occurs in the absence of the real stimuli that would normally precede the actual experience (Morris et al., 2005).

Moreover, as a potential psychological intervention, mantra meditation also differs from mindfulness-based interventions (MBIs) commonly used in sport contexts. Recent research defines mindfulness as “present-centered awareness of and bare attention to body sensations, affective valence, cognitive and emotional phenomena, and the external environment with an allowing and equanimous attitude” (Chems-Maarif et al., 2025, p. 1). Drawing on this definition, mindfulness-based interventions can be understood as structured psychological programmes designed to cultivate present-centred awareness across these experiential domains while fostering attitudes of acceptance and equanimity.

In contrast, mantra meditation requires individuals to repeatedly vocalise or silently repeat a word or phrase. For example, transcendental meditation is a standardised form of mantra meditation in which practitioners silently repeat a personally assigned mantra in order to facilitate entry into a state of restful awareness or “transcendence” (Ospina et al., 2007).

Despite these conceptual distinctions, the boundaries between mantra meditation and adjacent constructs are not consistently delineated in sport-related research, leading to potential conceptual ambiguity and misclassification. This lack of clarity limits the accumulation of coherent evidence and hinders theoretical development within sport psychology. Moreover, despite the well-documented benefits and diverse effects of mantra meditation on disease recovery and daily life in the non-athletic population, its application in sport settings remains relatively underexplored (Van Raalte et al., 2015). Taken together, these issues raise an important research question: how has mantra meditation been applied and conceptualised in sport? Therefore, the present study adopts a scoping review approach to map the existing literature on mantra meditation in sport, clarify its conceptual distinctions within the sport context, and identify directions for future research in this emerging area.

## Methods

### Operational definition

Conceptual distinctions outlined in the Introduction were translated into operational criteria to guide study selection, as detailed below.

#### Mantra meditation

For the purpose of this review, mantra meditation was operationally defined as a structured practice involving the intentional repetition (silent or vocalised) of a word, phrase, or sound to regulate attention or induce a meditative state. Studies were included if the practice was explicitly described as meditation or embedded within a formal meditative framework.

To ensure consistency in study selection, distinctions from related constructs were operationalised as follows:

#### Self-talk

Excluded when verbal repetition served performance, motivational, or instructional functions without a meditative or attentional regulation purpose.

#### Repetitive prayer

Included only when the practice emphasised attentional focus or meditative absorption; excluded when primarily devotional or communicative in nature.

#### Imagery

Excluded when cognitive rehearsal involved visualisation without repetitive verbal or sound-based focus.

#### Mindfulness

Excluded unless the intervention explicitly incorporated mantra repetition as a central attentional anchor.

#### Transcendental meditation

Included only when studies explicitly described mantra repetition as a central attentional mechanism; excluded when transcendental meditation was treated as a broader standardised programme without sufficient detail on mantra use.

### Search strategy

This study adhered to the Preferred Reporting Items for Systematic Reviews and Meta-Analyses extension for scoping reviews (PRISMA-ScR) guideline (Tricco et al., 2018). We employed a mixed retrieval strategy incorporating both electronic database searching and manual searching. Electronic databases included Web of Science, Psychology & Behavioural Sciences Collection, PubMed, SPORTDiscus, and Scopus. The search criteria covered titles, abstracts, and keywords, utilising searching keywords as follows: (“coping strateg*” OR “cognitive strateg*” OR “psychological strateg*” OR “self* regulation” OR “psychoso* intervention*” OR “psycholog* intervention*” OR “religious intervention” OR “spiritual intervention*” OR meditation* OR mindfulness* OR mantra* OR transcendent* OR “holy name repetition” OR “self* talk*” OR yoga*) AND (marathon* OR sport* OR athlet* OR runner* OR player* OR competit*). The search terms were expanded based on findings from previous evidence syntheses on mantra meditation in the general population (Hulett et al., 2023). The use of the asterisk as a wildcard aimed to broaden the search scope. Manual searching included both forward and backward citation tracking of the final studies identified through the electronic database search.

### Eligibility criteria

The inclusion criteria for this study comprised five key points: when classification was ambiguous, studies were included only if (1) repetition of a sound/phrase was central; (2) the stated purpose aligned with attentional regulation or meditative processes; (3) the subject of mantra implementation/practice is an athlete; (4) articles published in international journals in English; or (5) inclusion of articles across all publication years, with the final electronic database search conducted on 24 April 2025. Exclusion criteria encompassed three points: (1) grey literature (e.g., preprints, book chapters, theses, conference papers, and organisational reports); (2) articles with titles and abstracts presented in English but the main text written in a language other than English; or (3) mantra not associated with meditation (e.g., professional abbreviations such as “MANTRA: Method of Characteristics based Neutron Transport Code” in the field of nuclear engineering, or “MANTRA” referring to the Maudsley Anorexia Nervosa Treatment for Adults, a treatment protocol for anorexia nervosa).

### Literature screening

Literature screening involves four steps: 1) duplicate removal, 2) primary screening, 3) secondary screening, and 4) expert consensus. Duplicate removal was carried out in Zotero. The primary screening process included the independent assessment of titles, abstracts, and keywords by the first and second authors. Secondary screening involved an independent review of the full text of included articles from the primary screening result, with the same individuals conducting the screening. Any discrepancies in the inclusion outcomes during the primary and secondary screening process were resolved through discussions among all authors. Expert consensus was employed to review the results of the secondary screening. One expert has published in international sports psychology journals, while the other has experience conducting and publishing evidence syntheses in international journals. The experts did not independently determine study exclusion. Rather, they provided methodological advice regarding studies that required further consideration, particularly where the classification of the intervention as mantra meditation was unclear. Following this consultation, all authors collectively reviewed the study characteristics, intervention descriptions, and eligibility criteria before reaching a consensus decision. The final inclusion and exclusion decisions were therefore made by the author team rather than by the experts themselves.

### Quality assessment

The methodological quality appraisal used the Mixed Methods Appraisal Tool (MMAT, Hong et al., 2018). The MMAT includes two screening questions, five common study designs (i.e., qualitative, quantitative (randomised controlled trials), quantitative non-randomised, quantitative descriptive, and mixed methods), and a comments section. The screening questions (i.e., “Are there clear research questions?” and “Do the collected data allow addressing the research questions?”) are used to assess whether the included studies are empirical. If the answer to any of the screening questions is negative, the MMAT cannot be applied to the study. For each study design, five fundamental criteria are used to assess methodological quality. Evaluators are required to rate these criteria using three options (i.e., yes, no, or can’t tell), while comments provide evaluators with the opportunity to offer supplementary notes or explanations during the appraisal process. In this study, the evaluation was independently conducted by the first and second authors, with any discrepancies discussed and resolved through consensus among all authors. Although critical appraisal is not a mandatory component of scoping reviews (Tricco et al., 2018), we conducted a methodological quality assessment using the MMAT to provide readers with additional information about the methodological characteristics of the included studies. The appraisal was not used to rank the overall strength of evidence or to make claims about effectiveness; rather, it was used descriptively to support cautious interpretation of the available literature.

### Independent dual review process

Electronic searches, study selection, quality appraisal, data extraction, and synthesis were independently conducted by the first and second authors. Agreement regarding study inclusion based on electronic database searches was high (85.7%), calculated as the proportion of overlapping included studies relative to the average number of studies included by the two reviewers. Discrepancies in data extraction were resolved through discussion, with unresolved disagreements adjudicated by a third author.

### Risk of bias and publication bias

Methodological quality was assessed using MMAT, which is specifically designed for evidence syntheses. Given the methodological diversity of the included studies, a single appraisal tool was used to ensure comparability and coherence across study types. Publication bias was considered qualitatively by examining the predominance of positive findings, study sample sizes, and publication sources. This remains a potential limitation of the review.

## Results

An electronic and manual search yielded a total of 17,346 and 324 articles, respectively.

**Figure 1.**
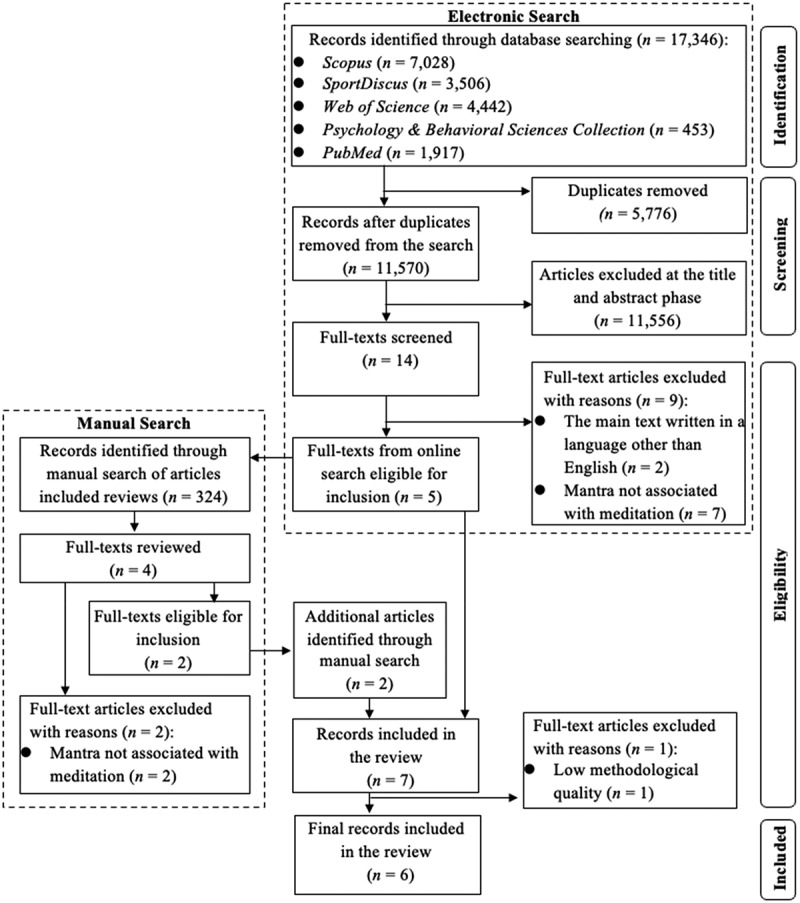
The PRISMA-ScR flow diagram.

Following the 3-step literature screening process (i.e., duplicate removal, primary screening, and secondary screening), a total of seven articles were initially included. After further review, one article from the initial list was excluded because the intervention description did not sufficiently meet the operational definition of mantra meditation used in this review. The expert consultation supported the authors’ reassessment of this borderline case, but the final exclusion decision was made by the author team based on the predefined eligibility criteria. The methodological quality appraisal was used to describe and contextualise the evidence base rather than as the primary basis for exclusion. As a result, this study included six articles for review (see Figure 1).

### Quality assessment

Table 1 presents the quality assessment of all included studies.

**Table 1.** MMAT quality appraisal of studies (chronological order).

Author(s)	Screening Questions		Qualitative					Quantitative (non-randomized)					Mixed Methods					Comments
Brick et al. (2015)	✓	✓	✓	✓	✓	✓	✓											This study meets all the criteria for MMAT assessment.
Van Raalte et al. (2015)	✓	✓	✓	✓	✓	×	✓											There is insufficient literature support that MM can assist runners in maintaining pace or rhythm, or serve other psychological purposes.
Kiemle-Gabbay (2017)	✓	✓	✓	✓	✓	–	–											A lack of explanation and literature support exists regarding whether MM is an effective coping strategy.
Cooper et al. (2019)	✓	✓	✓	✓	✓	✓	×											This study confused the concepts of MM and self-talk.
Samajdar and Mukherjee (2020)	✓	✓						–	✓	–	×	–						(1) The article did not provide information about the frequency of the MM intervention, the representativeness of the participants (e.g., age, gender, and level of physical activity), and potential confounding factors; (2) The article did not offer details regarding data completeness and the handling of missing data.
Voelker et al. (2021)	✓	✓	✓	✓	✓	✓	✓											This study meets all the criteria for MMAT assessment.

*Note*. ‘✓’ means that the criterion is met. ‘x’ means that the criterion is not met. ‘–’ means that there is not enough information in the paper to judge if the criterion is met or not; MM = Mantra meditation; The assessment of study designs for two columns (i.e., quantitative randomized controlled trials and quantitative descriptive), has been deleted because these types of study designs were not utilized in the included articles of this review.

*Screening questions 1: Are there clear research questions? Screening questions 2: Do the collected data allow addressing the research questions?

The five criteria for the qualitative studies are as follows: 1. Is the qualitative approach appropriate to answer the research question? 2. Are the qualitative data collection methods adequate to address the research question? 3. Are the findings adequately derived from the data? 4. Is the interpretation of results sufficiently substantiated by data? 5. Is there coherence between qualitative data sources, collection, analysis, and interpretation?

Five criteria for the quantitative (non-randomized) studies are as follows: 1. Are the participants representative of the target population? 2. Are the measurements appropriate in relation to both the outcome and the intervention (or exposure)? 3. Are the outcome data complete? 4. Are the confounders addressed in the study design and analysis? 5. Was the intervention administered (or exposure occurred) as intended during the study period?

Five criteria for the mixed methods of studies are as follows: 1. Is there an adequate rationale for using a mixed methods design to address the research question? 2. Are the different components of the study effectively integrated to answer the research question? 3. Are the outputs of the integration of qualitative and quantitative components adequately interpreted? 4. Are divergences and inconsistencies between quantitative and qualitative results adequately addressed? 5. Do the different components of the study adhere to the quality criteria of each tradition of the methods involved?

Based on the different applications of mantra, these articles were categorised into two domains: (1) mantra as a mental training strategy (*n* = 2) and (2) mantra as a coping strategy (*n* = 4). Table 2 summarises the findings from the included articles, presenting information on authors, study design, publication year, sample characteristics (e.g., gender, number of participants, level of sports, and type of sports), and conclusions on the application of mantra in the field of sports.

**Table 2.** The summary of findings from included articles (chronological order).

Author(s)	Year	Aim		Participant characteristics				Results
			Sample size	Gender	Type of sport	Sport level	Age	
Mantra as a Meditation Intervention (*n* = 2)								
Voelker et al.	2021	To examine the effectiveness of the Bodies in Motion program, including mantra meditation, in promoting a healthy self and body image among female student-athletes	*N* = 116	Female (*n* = 116)	Student-athletes (i.e., rowing/crew, cross-country, tennis, swimming/diving, gymnastics, riflery, track and field, soccer, basketball, figure skating, volleyball, golf, softball, skiing, ice hockey, and cheerleading)	College-level	Mean ± *SD* = 19.63 ± 1.23	The participants reported that through the Bodies in Motion program, they learned to use self-compassion mantra meditation to foster a healthier self-image and body image.
Samajdar and Mukherjee	2020	To assess the effect of mantra meditation on the attention, memory, and anxiety level of young athletes	*N* = 15	Not provided	Not provided	Not provided	Mean ± *SD* = 21.60 ± 1.84	Mantra meditation has a significant impact on the attention, memory, and anxiety level of athletes.
Mantra as a Coping Strategy (*n* = 4)								
Cooper et al.	2019	To identify primary influencers of the mental toughness variability of runners	*N* = 13	Male (*n* = 5), female (*n* = 8)	Runners	Elite	Mean ± *SD* = 48 ± 5.4	Runners may use mantra meditation practice as a coping strategy during competition to enhance their mental toughness.
Kiemle-Gabbay and Lavallee	2017	To examines student-athletes’ utilization of coping strategies to combat risk-taking, potential loss, trauma, and other stressors integral to their sport participation	*N* = 10	Male (*n* = 5), female (*n* = 5)	Student-athletes (i.e., skiers and snowboarders)	Semi-elite	18 to 24 (mean age = 21.2)	Athletes reported employing mantra meditation to cope with potential injury risks and pressures during their competitions.
Brick et al.	2015	To examine the situational factors that may influence cognitive strategy use by elite endurance runners.	*N* = 10	Male (*n* = 4), female (*n* = 6)	Endurance runners	Elite	Mean ± *SD* = 35.6 ± 6.6	Runners may engage in mantra practice as a self-regulatory strategy to cope with exertional pain.
Van Raalte et al.	2015	To explore the use of coping strategies by marathon runners in competition	*N* = 483	Male (*n* = 272), female (*n* = 211)	Marathon runners (including elite and nonelite runners)	Elite (*n* = 72); nonelite (*n* = 411)	18 to 78	Marathon runners report that they may use mantra practice during races to help maintain pace or rhythm and fulfill spiritual needs/ purposes.

*Note. SD* = Standard Deviation.

Table 2. *The Summary of Findings from Included Articles (Chronological Order)*

### Mantra meditation as a mental training strategy

In this domain, two studies were identified (Samajdar & Mukherjee, 2020; Voelker et al., 2021). These studies used mantra meditation as a mental training strategy and demonstrated its preliminary evidence in enhancing attention, memory, and body image while reducing anxiety among athletes.

One of the studies employed a prospective experimental design, with 45 athletes evenly distributed across three groups: experimental group 1, which received a normal meditation (15 minutes); experimental group 2, which received both a normal meditation and a mantra meditation (15 minutes); and a control group (Samajdar & Mukherjee, 2020). All subjects were assessed for attention, memory, and anxiety level at baseline and after three months. Results indicated that both meditation groups showed improvement in mental state compared to the control group, but experimental group 2 exhibited better improvement in attention and memory level, with a greater reduction in anxiety level. Thus, the authors concluded that normal meditation with mantra meditation can better impact athletes’ attention, memory, and anxiety levels.

In another study, Voelker et al. (2021) incorporated self-compassion mantra meditation into the Bodies in Motion programme and invited 116 female collegiate athletes to evaluate the perceived benefits of this programme in improving female athletes’ self-image through qualitative research. The findings indicated that, after participating in this programme, participants learned to use self-compassion mantra meditation to foster a healthier self-image and body image.

### Mantra meditation as a coping strategy

In this domain, four articles were identified (Brick et al., 2015; Cooper et al., 2019; Kiemle-Gabbay & Lavallee, 2017; Van Raalte et al., 2015). These studies explore athletes’ use of mantra meditation as a coping strategy (e.g., the cognitive strategy) during both competitions and training. The use of mantra in this context aims to enhance mental toughness, manage physical discomfort, cope with stress and risks, fulfil psychological needs, and maintain rhythm in sports.

For example, Van Raalte et al. (2015) surveyed 483 marathon runners on the use of coping strategies during prolonged competitions. They found that the majority of the participants (88%) reported using self-talk during marathon competitions. Among them, 11 non-elite runners reported using mantras (e.g., “Stride, stride, abide, abide”.) to maintain their pace or rhythm, or to serve a spiritual purpose. However, none of the elite athletes (*n* = 72) reported using mantras. This does not suggest that mantra meditation is only suitable for lower-level athletes, as participants in the other three included studies were all elite or semi-elite athletes from various sports, such as skiing and running (Brick et al., 2015; Cooper et al., 2019; Kiemle-Gabbay & Lavallee, 2017). These athletes also used mantra meditation as a coping strategy for a range of purposes.

For example, Kiemle-Gabbay and Lavallee (2017) explored the coping strategies that athletes may use to cope with potential injury risks and the stressors during sports participation. Semi-structured interviews with 10 semi-elite skiers and snowboarders revealed that two participants used mantras as coping strategies during sports participation. One snowboarder reported using the mantra (e.g., “It will be fine”.) when feeling the risk of injury during sports participation. Another participant reported using a mantra and repeatedly “touching wood” as a strategy to cope with competitive pressure.

In another interview-based study exploring the use of cognitive strategies among 10 elite endurance runners during competitions, Brick et al. (2015) found that athletes used mantra meditation as a positive cognitive strategy to cope with exertional pain and physical discomfort. Another study in this domain, conducted by Cooper et al. (2019), combined interviews and surveys to examine factors influencing mental toughness variability among elite track and field athletes during training and competition. One participant in their study reported using a mantra (e.g., “Be brave. Be strong. Be badass”.) to strengthen mental toughness during competitions.

## Discussion

This review aimed to explore the application of mantra meditation in the field of sport. Following the PRISMA-ScR guidelines, six relevant articles were included. Overall, these studies suggest that athletes have described using mantra practices both as a mental training strategy and as a coping strategy within sport contexts. Notably, the small number of eligible studies was not the result of overly restrictive inclusion criteria, as the review included studies from all publication years and employed a comprehensive search strategy across multiple databases. Rather, the limited evidence base appears to reflect the current state of research on mantra meditation in sport. Although mantra meditation has been more extensively investigated in other domains, relatively few studies have examined its application in sport-specific contexts. This scarcity of sport-specific research highlights an important knowledge gap and underscores the value of the present scoping review in mapping the existing literature and identifying priorities for future research. To further understand the potential mechanisms underlying these observations, the discussion first considers how mantra meditation may be interpreted through established theoretical frameworks in sport psychology.

### Theoretical integration with sport psychology frameworks

From a theoretical perspective, the potential benefits of mantra meditation in sport can be interpreted through the following three established frameworks in sport psychology, including self-regulation theory, attentional control theory, and stress and coping models. From a self-regulation perspective, optimal performance involves continuous monitoring of one’s behaviour and internal states, evaluation against personal standards, and subsequent adjustment of responses (Zimmerman, 2000). The rhythmic and repetitive nature of mantra meditation may provide a stable attentional anchor that facilitates sustained focus. Consequently, athletes may employ mantra repetition to regulate pacing, maintain rhythm, and preserve psychological stability during prolonged or high-pressure performances (Cooper et al., 2019; Kiemle-Gabbay & Lavallee, 2017; Van Raalte et al., 2015).

The potential effects of mantra meditation can also be interpreted through attentional control theory. According to this framework, anxiety can disrupt goal-directed attentional processes while increasing susceptibility to distracting or threat-related stimuli (Eysenck et al., 2007). Repetitive mantra use may help stabilise attentional focus by directing cognitive resources towards a simple and consistent internal cue, thereby reducing task-irrelevant thoughts and improving concentration during demanding athletic tasks. From this perspective, mantra repetition may partly explain why a participant in Kiemle-Gabbay and Lavallee (2017) reported using a mantra to cope with competitive pressure.

Within stress and coping models, mantra meditation may function as an emotion-focused coping strategy that assists athletes in regulating psychological responses to competitive stressors (Lazarus & Folkman, 1984). In situations involving fatigue, pain, injury risk, or performance pressure, mantra repetition may help athletes reinterpret or manage stressful experiences, thereby supporting emotional stability and psychological resilience. This perspective may also help explain why athletes report using mantra-based coping strategies when facing injury risk, exertional pain, or physical discomfort during sport participation (Brick et al., 2015; Kiemle-Gabbay & Lavallee, 2017).

**Figure 2.**
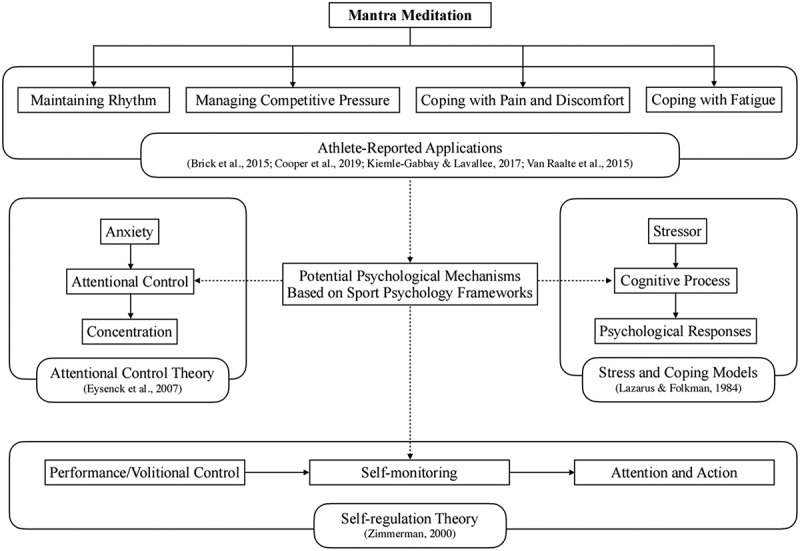
A theory-informed conceptual framework of athlete-reported applications of mantra meditation.

Taken together, these theoretical perspectives suggest that mantra meditation may influence athletic experiences through multiple psychological pathways (see Figure 2). Future research should employ controlled experimental designs to empirically test the effectiveness of mantra use in the performance contexts described by athletes, such as coping with competitive pressure, managing fatigue or pain, and maintaining rhythm during performance. Longitudinal research may further clarify under what conditions and for which athletes mantra-based strategies are most effective.

Beyond the theoretical insights discussed above, the findings of this review also highlight several conceptual and practical implications. First, this review identified a tendency in several studies to conflate mantra meditation with self-talk (Cooper et al., 2019, Kacperski et al., 2019; Kiemle-Gabbay & Lavallee, 2017; Van Raalte et al., 2015). However, these constructs are conceptually distinct in sport psychology. Self-talk generally refers to syntactically recognisable internal dialogue in which the sender and receiver of the message are the same, and it is often classified as positive, negative, instructional, or motivational (Van Raalte et al., 2016). In contrast, mantras may not carry explicit practical meaning and may serve spiritual, symbolic, ritualistic, or existential functions (Easwaran, 2013; Oman, 2025). For example, Van Raalte et al. (2015) reported that some marathon runners used mantras to fulfill spiritual needs rather than to provide motivational cues. Therefore, future research should carefully examine the purpose and meaning of repeated phrases used by athletes before classifying them as either mantra meditation or secular self-talk.

Second, within the domain of coping strategies, this review found that athletes who use mantra meditation during competitions are predominantly endurance athletes, such as marathon runners. This trend is supported by Van Raalte et al. (2016), who noted that mantra use may be particularly common among endurance athletes. One possible explanation is that endurance sports involve prolonged physical exertion, which increases the risk of fatigue and injury (Birat et al., 2020). As a result, these athletes may adopt mantra meditation to maintain a rhythmic pace, enhance mental toughness, and reduce injury risk (Brick et al., 2015; Cooper et al., 2019; Van Raalte et al., 2015).

Last, Van Raalte et al. (2015) found that only non-elite marathon runners reported using mantra meditation during competitions, leading the authors to suggest that mantra may be particularly beneficial for low-level endurance athletes. However, three other included studies documented the use of mantra among elite endurance athletes and semi-elite skiers (Brick et al., 2015; Cooper et al., 2019; Kiemle-Gabbay & Lavallee, 2017), indicating that mantra practices have been reported by athletes across different competitive levels. Despite these findings, Van Raalte et al. (2015) emphasised the need for further research to determine the effectiveness of mantra meditation in sports. This call is echoed in the current review, as much of the existing evidence is based on athletes’ personal accounts and experiential evidence (Brick et al., 2015; Cooper et al., 2019; Kiemle-Gabbay & Lavallee, 2017; Van Raalte et al., 2015), which may be influenced by subjective bias. To establish a more robust understanding, future research should examine, with more rigorous study designs, whether and how mantra meditation is associated with sport-relevant psychological and performance-related outcomes, such as attention, perceived stress, anxiety, fatigue, mental toughness, and athletic performance.

### Limitations and future directions

This review is subject to several limitations. First, only English-language publications were included, which may have resulted in the exclusion of relevant studies published in other languages and potentially limited the comprehensiveness of the review. Second, the review focused exclusively on athlete populations, which may restrict the generalisability of the findings to broader populations. Third, the evidence base identified in this review was small, with only six studies meeting the inclusion criteria. This limited number of studies restricts the strength and generalisability of the conclusions that can be drawn and highlights the early stage of research on mantra meditation in sport. Accordingly, the findings should be interpreted as exploratory and descriptive rather than as evidence of effectiveness. Caution is also warranted because the included studies varied in designs, populations, and applications.

Despite these limitations, the review highlights several important directions for future research. From a broader contemplative practice perspective, mantra meditation in sport may be understood as a form of meditation-based self-regulation. Unlike mindfulness meditation, which emphasises non-judgemental awareness of present-moment experiences, mantra meditation involves the repeated use of a word, phrase, or sound that may carry spiritual, symbolic, or personal meaning (Oman, 2025). However, the current evidence base remains limited, and the mechanisms through which mantra meditation may influence athletes’ psychological functioning are not yet well understood. Future studies are therefore needed to clarify its conceptual foundations, underlying mechanisms, and potential applications in sport settings.

In addition, although much of the existing intervention research on mantra meditation has focused on veterans with PTSD (Kelsven et al., 2024), no studies identified in the present review examined mantra meditation interventions among athletes experiencing PTSD-related symptoms. This gap may be particularly important given evidence suggesting that athletes, especially those recovering from serious injuries, may experience PTSD symptoms and other adverse psychological responses following injury (Alexander et al., 2024). Future research should therefore investigate the potential role of mantra meditation in supporting athletes experiencing PTSD-related symptoms, insomnia, and other psychological difficulties following sport-related injuries.

## Conclusion

This review synthesises existing evidence on the application of mantra practices in sport contexts. The included studies suggest that athletes use mantras as a psychological strategy for attentional regulation, coping with physical and psychological challenges, and maintaining pacing or rhythm during performance. In addition, some athletes described mantra use as supporting spiritual meaning and psychological well-being. However, most of the available evidence is derived from observational and qualitative studies, with only a limited number of intervention-based investigations. Therefore, the current findings should be interpreted as descriptive insights into athletes’ experiences rather than evidence of the effectiveness of mantra meditation. Future research should employ more rigorous experimental and longitudinal designs to examine the mechanisms, effectiveness, and potential applications of mantra meditation across diverse sports and athlete populations.
